# Differential gene transfers and gene duplications in primary and secondary endosymbioses

**DOI:** 10.1186/1471-2148-6-38

**Published:** 2006-04-26

**Authors:** Stefan Zauner, Peter Lockhart, Bettina Stoebe-Maier, Paul Gilson, Geoffrey I McFadden, Uwe G Maier

**Affiliations:** 1 Allan Wilson Centre for Molecular Ecology and Evolution, Institute of Molecular BioSciences, Massey University, Palmerston North, New Zealand; 2 Cell Biology, Philipps-University Marburg, Karl-von-Frisch Str., 35032 Marburg, Germany; 3 Walter and Eliza Hall Institute of Medical Research, Parkville, Victoria 3050, Australia; 4 Naturwissenschaffen, An der Zahlbach 15, 35039 Marburg, Germany; 5 Plant Cell Biology Research Centre, School of Botany, University of Melbourne, Victoria 3010, Australia

## Abstract

**Background:**

Most genes introduced into phototrophic eukaryotes during the process of endosymbiosis are either lost or relocated into the host nuclear genome. In contrast, *gro*EL homologues are found in different genome compartments among phototrophic eukaryotes. Comparative sequence analyses of recently available genome data, have allowed us to reconstruct the evolutionary history of these genes and propose a hypothesis that explains the unusual genome distribution of *gro*EL homologues.

**Results:**

Our analyses indicate that while two distinct *gro*EL genes were introduced into eukaryotes by a progenitor of plastids, these particular homologues have not been maintained in all evolutionary lineages. This is of significant interest, because two chaperone proteins always co-occur in oxygenic photosynthetic organisms. We infer strikingly different lineage specific processes of evolution involving deletion, duplication and targeting of *gro*EL proteins.

**Conclusion:**

The requirement of two *gro*EL homologues for chaperon function in phototrophs has provided a constraint that has shaped convergent evolutionary scenarios in divergent evolutionary lineages. G*ro*EL provides a general evolutionary model for studying gene transfers and convergent evolutionary processes among eukaryotic lineages.

## Background

Plastids, the solar powered energy factories of phototrophic eukaryotes, either translate mRNAs for their organelle-encoded genes or import nuclear-encoded proteins. In both cases, correct folding of proteins is managed by chaperones such as those of the GroEL family. These are an abundant class of chaperones, which are also found in mitochondria, hydrogenosomes and prokaryotes [1,2]. Their importance and distribution has led to intensive investigation of their function, and has culminated in the 'molecular chaperon concept' [3,4], which has strongly influenced current understanding of protein folding and assembly.

Early genomics on the cyanobacterium *Synechocystis *sp. PCC 6803 and on plastid chromosomes of eukaryotes has highlighted a surprisingly varied distribution of genome locations for GroEL homologues amongst photosynthetic taxa [5,6]. *Synechocystis *sp. PCC 6803 harbours two different *gro*EL genes, whereas only one is maintained in red algal plastomes and the plastid genome of the cyanelle. Genes encoding GroEL have not been located within the sequenced genomes of chloroplasts in green algae and land plants, but two nuclear homologs of *gro*EL, the *cpn*60s, have been detected in the nuclear genome of *Chlamydomonas reinhardii *and some land plants. In *Euglena gracilis*, no *gro*EL gene has been identified [7]. The secondary endosymbionts of a cryptomonad (*Guillardia theta*) and also a diatom (*Odontella sinensis*) are known to encode a single *gro*EL gene in their plastid, and it can be speculated that the presence of a single copy of *gro*EL may indicate the ancestral state in a primary endosymbiont [8,9]. Interestingly, the nucleomorph genome of the cyptomonad *Guillardia theta *harbours a *gro*EL homologue [6] and a *cpn*60-like homologue has recently also been found in the nucleomorph genome of another secondary endosymbiont: the chlorarachniophyte *Bigelowiella natans *(Gilson & McFadden, unpublished). Additionally, *cpn*60-like genes have recently been discovered in the nuclear genomes of other photosynthetic organisms: including in a red alga (*Cyanidioschyzon merolae*), a diatom (*Thalassiosira pseudonana*), and *Plasmodium falciparum *(a parasitic organism that harbours a degenerate plastid). With the exception of *P. falciparum*, the co-occurrence of two *gro*EL genes in the genomes of these recently sequenced organisms was predicted as necessary for maintaining chloroplast function [6]. We develop here a hypothesis for differential transfer and gene duplication that explains the distribution of *gro*EL homologues amongst the mulitple genomes of photosynthetic taxa. We discuss how these proteins may act as an important regulator for plastid functions.

## Results

### Substitution model selection

In all analyses, except analyses of the red/brown algal GroEL orthologues, an RtTEV model, that accommodated positional rate heterogeneity in some form was the model selected as best by ProtTest under the AIC criterion. In the case of the GroEL orthologues in red/brown algae, we noted that small differences in the AIC criterion separated a variety of different models. When an RtREV model was assumed, and positional rate heterogeneity was approximated by either a constant proportion of variable sites or a discrete gamma distribution of rate classes, we noticed that the optimal estimates for these parameters varied among evolutionary lineages (i.e different estimates were obtained for cyanobacterial GroEL1, cyanobacterial GroEL2, red algal/heterokont plastid GroEL1 like sequences, and highly diverged Cpn60-like sequences). When a uniform rate distribution was assumed, p_*var *_values ranged from 0.3 variable sites (cyanobacterial GroEL1) to 0.9 variable sites (highly diverged Cpn60-like sequences). When p_*var *_was set to 1, alpha shape parameter values ranged from 0.2 (for cyanobacterial GroEL1) to 0.8 (for highly diverged Cpn60-like sequences). We suggest that this degree of variation in parameter estimates for phylogenetic grouping of anciently diverged sequences is likely to reflect lineage specific differences in structural and functional constraints [10-12]. As such it is potentially problematic for phylogenetic reconstruction, since parallel increases in proportions of variable sites in different evolutionary lineages can sometimes induce a form of long branch attraction [10-12].

### Evolutionary tree building

**Figure 1:**
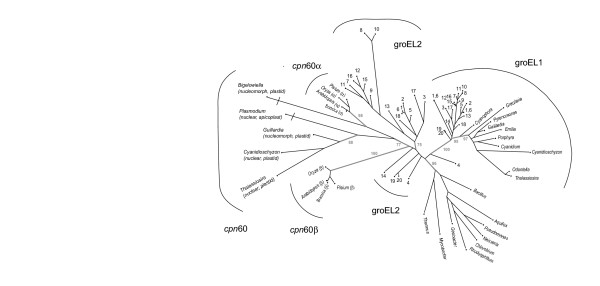
Optimal PhyML tree, built assuming an RtREV + G(α = 0.92) substitution model. Internal branches relevant to the discussion and which receive greater than 74% have been shown. Branch lengths are indicated. However, those subtending *Bigelowiella *and *Plasmodium *have been truncated. Cyanobacteria have been given number identifiers: [1] *Anabaena variabilis *29413, [2] *Anabaena *sp. strain L-31, [3] *Crocosphaera watsonii *WH 8501, [4] *Gloeobacter violaceus *PCC 7421, [5] Nostoc punctiforme ATCC 29133, [6] *Anabaena *sp. PCC 7120, [7] *Prochlorococcus marinus *subsp. *marinus *str. CCMP1375, [8] *Prochlorococcus marinus *str. MIT 9312, [9]*Prochlorococcus marinus *str. MIT9313, [10] *Prochlorococcus *pastoris str. CCMP1986, [11] *Prochlorococcus marinus *str. NATL2A, [12] Synechococcus sp. CC9605, [13] *Synechococcus elongatus *7942, [14] *Synechococcus vulcanus*, [15] *Synechococcus sp*. wh8102, [16] *Synechococcus *sp. CC9902, [17] *Synechocystis *PC 6803, [18] *Trichodesmium erythraeum *IMS101, [19] Cyanobacteria bacterium Yellowstone A-Prime, and [20] Cyanobacteria bacterium Yellowstone B-Prime.

Optimal PhyML (protein maximum likelihood) trees showed similar topological relationships over a wide range of p_*var *_values specified to accommodate positional rate heterogeneity. The robustness of phylogenetic relationships to sampling error was also found to be relatively stable, when evaluated using non-parametric bootstrapping (100 replicates). Figure 1 shows the optimal unrooted phylogenetic tree built assuming an RtREV model with positional rate heterogeneity modelled with a discrete gamma distribution (α = 0.92; 4 discrete rate categories).

Although, it contains more GroEL homologues than was available to Wastl et al. [6], our phylogenetic reconstruction here is nevertheless consistent with observations and inferences made by Wastl et al. [6]. Figure 1 shows that (a) the *gro*EL1 like genes from the plastid genome of red algae/heterokonts and cryptophytes are most closely related to the cyanobacterial *gro*EL1 genes. (b) Interestingly the cyanelle *gro*EL1 like homologue is somewhat intermediate between cyanobacterial and red algal/chromist *gro*EL1-like sequences, a finding that may reflect the cyanobacterial-like nature of this plastid. (c) The non-photosynthetic eubacterial *gro*EL sequences are arguably more similar to the *gro*EL2 sequences found in cyanobacteria. In any event, the *gro*EL2 orthologues in cyanobacteria are genetically more diverse than the *gro*EL1 sequences in the same taxa. These observations may suggest that *gro*EL2 orthologues represent an ancestral from of GroEL. However, our inference that structural/functional constraints differ amongst GroEL homologues, means that it is not possible to exclude other interpretations. (d) Assuming the root of the tree joins the branch leading to the non-photosynthetic taxa, Figure 1 places the *Gloeobacter *"A" and "B" sequences as the ancestral forms of GroEL homologues in photosynthetic taxa. (e) In the nuclear genomes of a diatom (*Thalassiosira pseudonana*), an alveolate (*Plasmodium falciparum*), the higher plants and the nucleomorph genomes of a chlorarachniophyte (*Bigelowiella natans*), a cryptophyte (*Guillarida theta*) and a red algae (*Cyanidoschyzon merolae*) *gro*EL2 type sequences are found. In the case of higher plants, two forms of groEL2 occur, and appear to represent forms of groEL duplicated from an ancestral groEL2-like sequence. Inferences concerning the origin of this duplication are potentially problematic. A relatively high bootstrap value in Figure 1 (77%) suggests that it may have occurred prior to the divergence of plastids. However, potential long branch attraction problems concerning the placement of α and β Cpn60-like sequences from eukaryotes make this conclusion tentative. Gene duplication within the green lineage and differences in the functional/structural constraints of green α and β Cpn60-like sequences might also explain the results observed Although *Chlamydomonas reinhardtii *Cpn60 α and β sequences (AAA98642 and AAA98643) are not included in our phylogenetic analysis shown in Figure 1 (because their inclusion significantly reduced the alignment length, and increased phylogenetic uncertainty). However, it is clear from other phylogenetic analyses (unpublished) that this green alga contains the two forms of *gro*EL2 also present in higher plants.

## Discussion

### Hypotheses of origin

Our phylogenetic reconstruction suggests a complex pattern of genome transfers, losses and duplications in the evolution of *gro*EL sequences from photosynthetic taxa. In the earliest cyanobacterial-like prokaryotes, the ancestral *gro*EL sequence appears to have duplicated to form an "A" and a "B" type sequence that is still present today in the genome of *Gloeobacter *[14,15]. It appears that the "A" (*gro*EL2-like) and "B"(more *gro*EL1-like) forms have been inherited by most cyanobacteria, and also the endosymbiont(s) involved in primary plastid endosymbiosis. During the process of endosymbiosis, the *gro*EL2-like orthologue has been transferred from the endosymbiont genome to the nuclear genome in heterokonts, red algae, green algae and high plants, and cryptophytes. We predict that this also will be true for haptophytes and glaucocystophytes. Less certain may be prediction of the pattern of evolution in peridinin-containing dinoflagellates, which in other respects appear highly distinctive in their evolution [16,17]. An interesting observation is that *Cyanophora paradoxa*, the molecular prototype of the glaucophytes, shows not only archaic features by encoding a *gro*ES in its plastid genome, but also by containing a *gro*EL1 sequence, somewhat intermediate in sequence identity between cyanobacterial and plastid encoded *gro*EL1 genes. In the case of the chlorophyte lineage, it appears that it has been the *gro*EL1 form that has been lost, and in *Chlamydomonas *and higher plants, this has been replaced by a duplicated form of a *gro*EL2-like sequence. This situation may also be true for the endosymbiont of the chlorarachniophyte *B. natans *(generally assumed to have been a green alga [16]), since phylogenetic reconstructions, including those with partial *Chlamydomonas *GroEL sequences (not shown), provide some support for *Bigelowiella *and cpn60α containing taxa being most closely related (e.g. Figure 1). Several investigations on the evolution of the *Plasmodium *apicoplast indicate a red algal origin for this organelle [18,19]. If so, then based on the generalisations stated above, one would expect that the *gro*El/*cpn*60 distribution should be similar to the situation found in heterokonts. Additional duplications, of *gro*EL1 genes (in the presence of *gro*EL2) have also occurred in some species of filamentous cyanobacteria and this observation is discussed in the following section.

### GroEL proteins and protein folding

In all eukaryotes and prokaryotes that carry out oxygenic photosynthesis, and for which genome data is complete, two different forms of *gro*EL/*cpn*60 are known to exist. Thus, at least two copies of the gene or protein seem to be necessary for a complete chaperon function. Further, the importance of having two divergent forms of GroEL for protein folding is suggested from observations on GroEL sequences in *Synechocystis *sp. PCC 6803, where it has been observed that GroEL1 and GroEL2 respond differently to heat shock and light conditions [20,21]. An interesting speculation is that the number and genetic diversity of GroEL homologues reflects complexity of morphotype in cyanobacteria. This is suggested from comparison within filamentous cyanobacteria. Those strains that harbour three *GroEL *genes exhibit complex developmental stages (akinetes and hormogonia) whereas filamentous strains with a simpler morphotype, such as *Nostoc *sp. PCC 7120, harbour only two *GroEL *genes. Further study is needed to evaluate whether particular *gro*EL homologues are specifically expressed in different developmental stages. Interestingly, *P. falciparum *encodes one *cpn*60 gene, but no *gro*EL1 in the apicoplast genome. If phylogenetic inferences of a close relationship with red algae [18,19] are correct, then this observation may reflect relaxed constraints for protein folding for proteins of the apicoplast, and this speculation is also worth further investigation.

## Conclusion

Although, phylogenetic reconstruction of individual gene histories is inherently problematic for anciently diverged taxa [5,10,11] phylogenetic reconstruction for *gro*EL homologues nevertheless provides a framework for developing understanding of genome-wide patterns of gene loss, relocation and multiple events of gene duplication. Our results presented here support and extend the hypothesis of *gro*EL/*cpn*60 evolution by Wastl et al. [6] which suggests a pattern of differential serial gene transfer and gene duplication.

## Methods

### Resources for sequences

Cyanobacterial *gro*EL genes were retrieved from Genbank and the cpnDB chaperonin sequence database [22]. Two *gro*EL genes were found to be present in *Synechocystis *sp. PCC 6803. One of these genes, termed *gro*EL1 (slr2076), is arranged in an operon together with *gro*ES, whereas the other, *gro*EL2 (sll0416), is not adjacent to a small subunit gene [23]. In comparing homologues from other cyanobacteria to those of *Synechocystis *sp. PCC 6803, we have adopted the terminology of "*gro*EL1" and "*gro*EL 2". All unicellular forms of cyanobacteria retrieved from database searches were found to contain two *GroEL *genes. In contrast, filamentous strains showed variation in the number of *gro*EL genes. *Nostoc punctiforme *and *Anabaena variabilis *ATCC29413 harbour three *gro*EL genes, whilst *Nostoc *sp. PCC 7120 with two *gro*EL genes, was similar to that of unicellular forms. Genbank also provided us with several entries for *cpn*60 from several land plants, *Chlamydomonas reinhardii *and the nucleomorph of the cryptophyte *Guillardia theta*. In a recently finished genome project on the nucleomorph genome of the chlorarachniophyte *Bigelowiella natans *(Gilson & McFadden, unpublished), a further nucleomorph-encoded *cpn*60 gene has been annotated. Two new genome projects on *Cyanidioschyzon merolae *[24] and *Thalassiosira pseudonana *[25] were also the source for additional nuclear-located *cpn*60 genes. In PlasmoDB [26], the genome data base for *Plasmodium falciparum*, two different *cpn*60 genes have been annotated in the nuclear genome [27], one encodes a mitochondrial, and the other an apicoplast targeted copy [28]. Additional, BLAST searches of NCBI were made against available plastid genomes using cyanobacterial *gro*EL genes. Significant hits were obtained with the plastid genomes from red (*Gracilaria verrucosa*, *Cyanidium caldarium*, *Porphyra purpurea*, *Cyanidioschyzon merolae*) and secondary red plastids (*Odonetella sinensis*, *Guillardia theta*, *Pyrenomonas salina*, *Thalassiosira pseudonana*). These sequences, together with the nuclear, nucleomorph, plastid, and cyanobacterial sequences were aligned using the progressive alignment procedure implemented in CLUSTALX [29], edited to remove any ambiguously aligned regions, and conserved blocks of aligned residues (containing 495 amino acids) were used for phylogenetic analyses. The aligned data matrix and accession details are available from the authors on request.

### Substitution model selection

The online version of ProtTest v1.2.6 [30,31], implementing the Akaike Information criterion (AIC) was used to select the most appropriate amino acid substitution models for tree building analyses. The cyanobacteria *GroEL*1 and *GroEL*2 datasets used for estimating parameters had identical taxon sampling (10 taxa: *Synechocystis *PC 6803, *Crocosphaera watsonii *WH 8501, *Trichodesmium erythraeum *IMS101, *Synechococcus elongatus *7942, *Synechococcus vulcanus*, *Synechococcus sp*. wh8102, *Prochlorococcus marinus *str. MIT9313, *Prochlorococcus marinus *str. CCMP1375, *Prochlorococcus *pastoris str. CCMP1986). Estimates were also made for bacteria (9 taxa: *Thermus thermophilus, Rhodospirillum rubrum, Neisseria meningitidis, Geobacter metallireducens, Pseudomonas aeruginosa, Mycobacterium avium, Bacillus subtilis, Aquifex aeolicus *and *Chlorobium tepidum*; and for red/brown algal chloroplast located orthologues (8 taxa: *Guillardia theta*, *Pyrenomonas salina*, *Cyanidium caldarium*, *Porphyra purpurea*, *Gracilaria tenuistipitata *var. liui, *Odontella sinensis*, *Thalassiosira pseudonana *and *Cyanidioschyzon merolae*) and also for highly diverged Cpn60-like sequences (5 taxa: *Thalassiosira pseudonana*, *Cyanidioschyzon merolae*, *Plasmodium falciparum, Bigelowiella natans *and *Guillardia theta*).

### Evolutionary tree building

Trees were reconstructed from amino acid sequences using the windows version of PhyML [32]. Trees were built assuming an RTRev model and the optimal tree shown in Figure 1 displayed using SplitsTree4.0 [33]. The robustness of phylogenetic reconstructions to variations in assumptions of positional rate heterogeneity was investigated by assuming (a) different proportions of variable sites (p_*var *_range = 0.3–1.0) and (b) a discrete gamma distribution of rate classes and a range of alpha shape parameter values (0.2–1). Non-parametric bootstrap trees were analyzed to assess the significance of sampling variability.

## Authors' contributions

**Figure 2:**
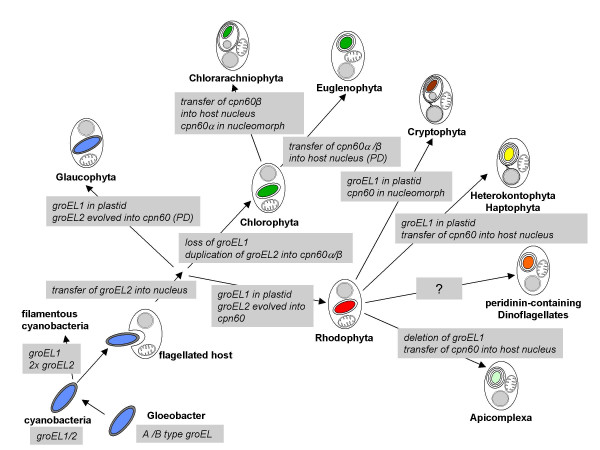
Scheme depicting an evolutionary hypothesis that explains genome locations of *gro*EL and *cpn*60. An ancestral eubacterial-like *gro*EL duplicated in cyanobacteria to give two homologues: *gro*EL1 and *gro*EL2. Both copies were inherited by phototrophic eukaryotes. One of the copies, *gro*EL1, has been lost from the plastid genome in some lineages. *gro*EL2 was transferred into the cell nucleus and gave rise to *cpn*60 in the case where *gro*EL1 is still maintained in the plastid genome, or into cpn60α and *cpn*60β, where *gro*EL1 has been deleted. For details see text. P.D. = predicted.

PG and GIM sequenced and analysed *cpn*60 from *Bigelowiella natans*, and contributed to the manuscript. BSM has drawn Figure 2 and contributed to the manuscript. PJL analysed the substitution properties of the data, constructed the phylogenetic trees and wrote together with UGM the manuscript. Data mining was done by SZ, who contributed to the manuscript as well. UGM has initiated the project. All authors read and approved the final manuscript.

## References

[B1] Bui ET, Bradley PJ, Johnson PJ (1996). A common evolutionary origin for mitochondria and hydrogenosomes. Proc Natl Acad Sci U S A.

[B2] Walter S (2002). Structure and function of the GroE chaperone. Cell Mol Life Sci.

[B3] Ellis RJ, van der Vies SM (1991). Molecular chaperones. Annu Rev Biochem.

[B4] Ellis RJ (2003). Protein folding: importance of the Anfinsen cage. Curr Biol.

[B5] Martin W, Stoebe B, Goremykin V, Hansmann S, Hasegawa M, Kowallik KV (1998). Gene transfer to the nucleus and the evolution of chloroplasts. Nature.

[B6] Wastl J, Fraunholz M, Zauner S, Douglas S, Maier UG (1999). Ancient gene duplication and differential gene flow in plastid lineages: the GroEL/Cpn60 example. J Mol Evol.

[B7] Hallick RB, Hong L, Drager RG, Favreau MR, Monfort A, Orsat B, Spielmann A, Stutz E (1993). Complete sequence of *Euglena gracilis *chloroplast DNA. Nucleic Acids Res.

[B8] Kowallik KV, Stoebe B, Schaffran I, Kroth-Pancic PG, Freier U (1995). The chloroplast genome of a chl a+c-containing alga, *Odontella sinensis*. Plant Mol Biol Reporter.

[B9] Douglas SE, Penny SL (1999). The plastid genome of the cryptophyte alga, *Guillardia theta*: complete sequence and conserved synteny groups confirm its common ancestry with red algae. J Mol Evol.

[B10] Lockhart PJ, Novis P, Milligan BG, Riden J, Rambaut A, Larkum AWD (2005). Heterotachy and tree building: a case study with plastids and eubacteria. Mol Biol Evol.

[B11] Lockhart PJ, Steel MA (2005). A tale of two processes. Syst Biol.

[B12] Guo Z, Stiller JW Comparative genomics and evolution of proteins associated with RNA polymerase II C-terminal domain. Mol Biol Evol.

[B13] Holland BR, Huber KT, Moulton V, Lockhart PJ (2004). Using consensus networks to visualize contradictory evidence for species phylogeny. Mol Biol Evol.

[B14] Nakamura Y, Kaneko T, Sato S, Mimuro M, Miyashita H, Tsuchiya T, Sasamoto S, Watanabe A, Kawashima K, Kishida Y, Kiyokawa C, Kohara M, Matsumoto M, Matsuno A, Nakazaki N, Shimpo S, Takeuchi C, Yamada M, Tabata S (2003). Complete genome structure of *Gloeobacter violaceus *PCC a cyanobacterium that lacks thylakoids. DNA Res.

[B15] Nakamura Y, Kaneko T, Sato S, Mimuro M, Miyashita H, Tsuchiya T, Sasamoto S, Watanabe A, Kawashima K, Kishida Y, Kiyokawa C, Kohara M, Matsumoto M, Matsuno A, Nakazaki N, Shimpo S, Takeuchi C, Yamada M, Tabata S (2003). Complete genome structure of *Gloeobacter violaceus *PCC 7421, a cyanobacterium that lacks thylakoids (supplement). DNA Res.

[B16] Stoebe B, Maier UG (2002). One, two, three: nature's tool box for building plastids. Protoplasma.

[B17] Keeling PJ (2004). Diversity and evolutionary history of plastids and their hosts. Amer J Bot.

[B18] Foth BJ, McFadden GI (2003). The apicoplast: a plastid in *Plasmodium falciparum *and other Apicomplexan parasites. Int Rev Cytol.

[B19] Coppin A, Varre JS, Lienard L, Dauvillee D, Guerardel Y, Soyer-Gobillard MO, Buleon A, Ball S, Tomavo S (2005). Evolution of plant-like crystalline storage polysaccharide in the protozoan parasite *Toxoplasma gondii *argues for a red alga ancestry. J Mol Evol.

[B20] Glatz A, Horvath I, Varvasovszki V, Kovacs E, Torok Z, Vigh L (1997). Chaperonin genes of the *Synechocystis *PCC 6803 are differentially regulated under light-dark transition during heat stress. Biochem Biophys Res Commun.

[B21] Kovacs E, van der Vies SM, Glatz A, Torok Z, Varvasovszki V, Horvath I, Vigh L (2001). The chaperonins of *Synechocystis *PCC 6803 differ in heat inducibility and chaperone activity. Biochem Biophys Res Commun.

[B22] Hill JE, Penny SL, Crowell KG, Goh SH, Hemmingsen SM (2004). cpnDB: a chaperonin sequence database. Genome Res.

[B23] Kaneko T, Sato S, Kotani H, Tanaka A, Asamizu E, Nakamura Y, Miyajima N, Hirosawa M, Sugiura M, Sasamoto S, Kimura T, Hosouchi T, Matsuno A, Muraki A, Nakazaki N, Naruo K, Okumura S, Shimpo S, Takeuchi C, Wada T, Watanabe A, Yamada M, Yasuda M, Tabata S (1996). Sequence analysis of the genome of the unicellular cyanobacterium *Synechocystis *sp. strain PCC6803. II. Sequence determination of the entire genome and assignment of potential protein-encoding regions. DNA Res.

[B24] Matsuzaki M, Misumi O, Shin IT, Maruyama S, Takahara M, Miyagishima SY, Mori T, Nishida K, Yagisawa F, Nishida K, Yagisawa F, Nishida K, Yoshida Y, Nishimura Y, Nakao S, Kobayashi T, Momoyama Y, Higashiyama T, Minoda A, Sano M, Nomoto H, Oishi K, Hayashi H, Ohta F, Nishizaka S, Haga S, Miura S, Morishita T, Kabeya Y, Terasawa K, Suzuki Y, Ishii Y, Asakawa S, Takano H, Ohta N, Kuroiwa H, Tanaka K, Shimikzu N, Sugano S, Sato, Nozaki H, Ogasawara N, Kohara Y, Kuroiwa T (2004). Genome sequence of the ultrasmall unicellular red alga *Cyanidioschyzon merolae *10D. Nature.

[B25] Armbrust EV, Berges JA, Bowler C, Green BR, Martinez D, Putnam NH, Zhou S, Allen AE, Apt KE, Bechner M, Brezinski MA, Chaal BK, Chiovitti A, Davis AK, Demarest MS, Detter JC, Glavina T, Goodstein D, Hadi MZ, Hellstein U, Hildebrand M, Jenkins BD, Jurka J, Kapitonov VV, Kroger N, Lau WW, Lane TW, Larimer FW, Lippmeier JC, Lucas S, Medina M, Montsant A, Obornik M, Parker MS, Palenik B, Pazour GJ, Richardson PM, Rynearson TA, Saito MA, Schwartz DC, Thamatrakoln K, Valentin K, Vardi A, Wilkerson FP, Rokhsar DS (2004). The genome of the diatom *Thalassiosira pseudonana*: ecology, evolution, and metabolism. Science.

[B26] http://www.plasmoDB.org.

[B27] Gardner MJ, Hall N, Fung E, White O, Berriman M, Hyman RW, Carlton JM, Pain A, Nelson KE, Bowman S, Paulsen IT, James K, Eisen JA, Rutherford K, Salzberg SL, Craig A, Kyes S, Chan MS, Nene V, Shallom SJ, Suh B, Peterson J, Angiuoli S, Pertea M, Allen J, Selengut J, Haft D, Mather MW, Vaidya AB, Martin DM, Fairlamb AH, Fraunholz MJ, Roos DS, Ralph SA, McFadden GI, Cummings LM, Subramanian GM, Mungall C, Venter JC, Carucci DJ, Hoffman SL, Newbold C, Davis RW, Fraser CM, Barell B (2002). Genome sequence of the human malaria parasite *Plasmodium falciparum*. Nature.

[B28] Sato S, Wilson RJ (2004). The use of DsRED in single- and dual-color fluorescence labeling of mitochondrial and plastid organelles in *Plasmodium falciparum*. Mol Biochem Parasitol.

[B29] Thompson JD, Gibson TJ, Plewniak F, Jeanmougin F, Higgins DG (1997). The CLUSTAL_X windows interface: flexible strategies for multiple sequence alignment aided by quality analysis tools. Nucleic Acids Res.

[B30] http://darwin.uvigo.es/.

[B31] Abascal F, Zardoya R, Posada D (2005). ProtTest: Selection of best-fit models of protein evolution. Bioinformatics.

[B32] http://atgc.lirmm.fr/phyml/.

[B33] Huson DH, Bryant D (2006). Application of phylogenetic networks in evolutionary studies. Mol Biol Evol.

